# Influence of Access Cavity Design on the Fracture Strength of Endodontically Treated Teeth Restored Using Short Fiber-Reinforced Composite and High Strength Posterior Glass Ionomer Cement

**DOI:** 10.7759/cureus.28135

**Published:** 2022-08-18

**Authors:** Vaddempudi Divyasree, JMV Raghavendra Reddy, Veeramachaneni Chandrasekhar, Swetha Kasam, Nimeshika Ramachandruni, Sivaram Penigalapati, Swathi Aravelli, Sindhura Alam

**Affiliations:** 1 Conservative Dentistry and Endodontics, Malla Reddy Dental College for Women, Hyderabad, IND

**Keywords:** ninja access cavity, conservative access cavity, truss access cavity, peri-cervical dentine, short fiber-reinforced composite

## Abstract

Aim: This in vitro study aimed to determine the influence of access cavity design and residual tooth structure and to compare the fracture resistance of the teeth post endodontically restored with short fiber-reinforced composite (GC everX Posterior; GC, India) and conventional posterior high-strength GIC (Glass Ionomer Cement) (GC Gold Label IX; GC, India).

Methods: Ninety extracted human mandibular molars were classified into five groups, i.e., one control group (n = 10) and four test groups based on the access cavity design (n = 20): Traditional access cavity (TAC), Conservative access cavity (CAC), Ninja access cavity (NAC), and Truss access cavity (TRAC). Then 80 teeth in test groups were endodontically treated and further subdivided (n = 10) based on post-endodontic restorative materials, i.e., short fiber-reinforced composite (SFC) and Type 9 GIC. Samples were then subjected to fracture resistance under a universal testing machine and fracture loads were compared statistically.

Results: The fracture resistance of various access cavity designs (TAC, CAC, NAC, and TRAC) varied significantly (P < .05). Regardless of access cavity design, teeth restored with SFC had higher fracture resistance than teeth restored with high strength posterior GIC (P = .001).

Conclusion: Using newer access cavity designs like (CAC, NAC, and TRAC) and reinforcing the teeth with a post-endodontic restoration such as SFC, fracture resistance of endodontically treated teeth can be improved notably.

## Introduction

Endodontic treatment aims to save teeth with damaged and infected pulp that would otherwise be lost or extracted. The amount of tooth material lost is one of the key reasons that the teeth that have been endodontically treated are brittle and prone to fracture. Extensive preparation of endodontic access cavities minimizes the level of healthy dentin and promotes the tooth's deformability [1-3]. Clark and Khademi have developed a novel approach to cavity preparation termed minimally invasive access, inspired by the concept of minimally invasive dentistry [4]. It prioritizes the preservation of peri-cervical dentin and also a section of the pulp chamber roof known as "Soffit," and the design of the access cavity was termed "conservative access cavity" (CAC). By designing "Ninja" and "Truss" access cavities (NACs and TRACs), a few endodontists have accentuated this concept. The Ninja access cavity (NAC) is a compact hole on the occlusal surface that enables the dentist to locate and access all canal orifices [5]. The truss access cavity (TRAC), on the other hand, offers direct access to every canal orifice from the occlusal surface, eliminating the need to remove the entire pulp chamber roof [6].

Intracoronary tooth reinforcement is vital to protect teeth against fracture, specifically in posterior teeth where occlusal forces can cause cusps to fracture if they are not protected. As a result, root canal therapy should not be deemed complete until a suitable post-endodontic restoration has been placed. For endodontically treated teeth, an ideal final restoration should retain aesthetics, conserve remaining tooth structure, aid proper functioning, and minimize microleakage [7]. Generally, materials like amalgam, Type-9 GIC (Glass Ionomer Cement), and composites are used for post-endodontic restoration in regular clinical practice. SFC (short fiber-reinforced composite) is a new Type of dental restorative material. These composite resins are designed to be used in areas that are subjected to high levels of stress, such as molars. When compared to traditional particulate-filled composite resins, these composite resins were augmented with short E-glass fiber fillers, which significantly improved load-bearing capacity, flexural strength, and fracture toughness. Short fiber-reinforced composite (SFC) resin also demonstrated control of polymerization shrinkage stress by fiber orientation, resulting in decreased marginal microleakage when compared to traditional ones [8-10].

The aim of this in vitro study was to determine the influence of access cavity design and residual tooth structure and to compare the fracture resistance of the teeth post endodontically restored with short fiber-reinforced composite (GC everX Posterior; GC, India) and conventional posterior high-strength GIC (GC Gold Label IX; GC, India).

## Materials and methods

After obtaining ethical clearance from the Institutional Ethical Committee, Malla Reddy Dental College For Women (#MRDCW/IEC/AP/19/2022), a total of 90 previously extracted, sound, non-carious human mandibular molar teeth with identical anatomical features for various reasons were chosen. Teeth were debrided and placed in sequentially numbered containers containing 0.9 percent saline solution until use. The teeth were divided into five groups at random: Group 0: Intact teeth (control group) (n=10); Group 1: Traditional access cavity (TAC) (n = 20); Group 2: Conservative access cavity (CAC) (n = 20); Group 3: Ninja access cavity (NAC) (n = 20); and Group 4: Truss access cavity (TRAC) (n = 20). Before the access cavity preparation, outlines of the various access cavity designs were drawn on the teeth of each group with a black marker pen, and then #856 diamond burs placed on a high-speed handpiece with water coolant were used to drill access cavities in all of the teeth.

**Figure 1:**
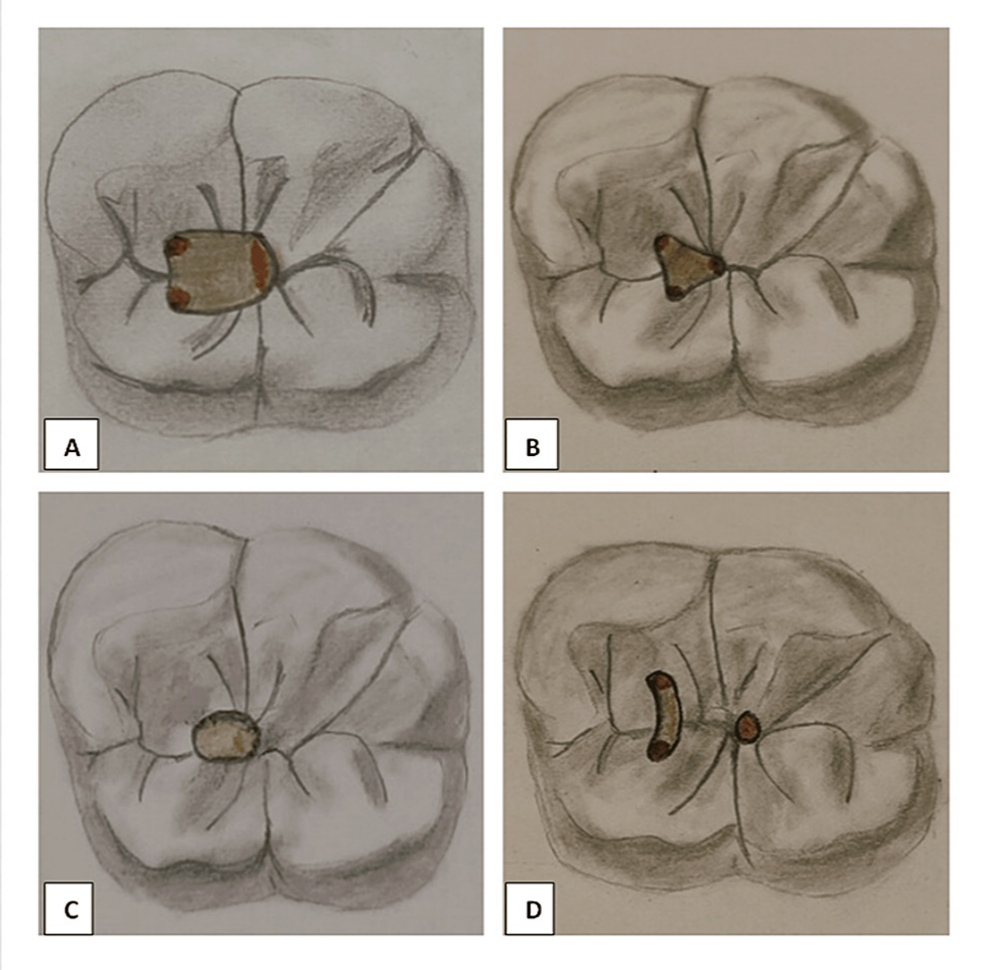
Cavity designs in a mandibular first molar from occlusal view (A) Traditional, (B) Conservative, (C) Ninja, and (D) Truss access cavity designs in a mandibular first molar from occlusal view

In the TAC group, teeth were accessed following the concepts reported formerly (Figure 1A) [11]. In the CAC group, teeth gained access to the mesial quarter of the central fossa and enlarged distally and apically while retaining a portion of the pulp chamber roof. All of the canal orifices are located at the same visual angulations, and circumferential peri-cervical dentin amputation was reduced to preserve a portion of the pulp chamber roof (Figure 1B). The NAC was opened perpendicularly at the deepest point of the occlusal surface with a # 1014 diamond round bur. The cavity was then slightly widened buccolingually with a fissure bur when the pulp chamber was reached. The cavity's mesiodistal and buccolingual lengths were fixed at 2 and 3 mm, respectively [8]. In the occlusal plane, an oblique projection towards the central fossa of the root canal orifices produced the "Ninja" access outline (Figure 1C). Truss access cavity (TRAC) preparations were carried out by using cautious opening procedures to keep a portion of the pulp chamber roof intact. To appropriately orient the bur, it was required to employ an X-ray probe (UNC 15) to gauge the length between the marginal ridges or buccal/lingual aspects of teeth and the perpendicular projections to the occlusal surface of their canal orifices. In the buccal-lingual direction, single access to the mesial canals was established, accompanied by circular access to the distal canal orifice. The single oval access to the mesial canals was obtained by uniting the two access slots formed following the perpendicular projection to the occlusal surface of the mesial canals and increasing them up to 1.2 mm for the oval optimum diameter; the circular access to the distal canal began with a single access slot formed following the perpendicular projection to the distal canal's occlusal surface, and it was then extended circularly to a diameter of 1.2 mm (Figure 1D). A 10 K-file was utilized to negotiate root canals until the tip was seen on the apical foramen, and the working length was established to be 1.0 mm shorter. ProTaper Universal Rotary (DENTSPLY, Switzerland) was used to prepare the root canals up to size F3 (25 mm). Canals were irrigated with 5 ml of 5.25% sodium hypochlorite and 5 ml of 17% ethylene diamine tetraacetic acid (EDTA), followed by final irrigation with saline. Obturation was performed with ProTaper universal F3 GP cones (DENTSPLY, Switzerland) using zinc oxide eugenol root canal sealer. Following the obturation, teeth were subdivided into two groups based on the post-endodontic restorative material, i.e., Type 9 GIC (GC Gold Label IX Extra Posterior GI Cement; GC, India) and short fiber-reinforced composite (GC everX Posterior; GC, India).

**Figure 2:**
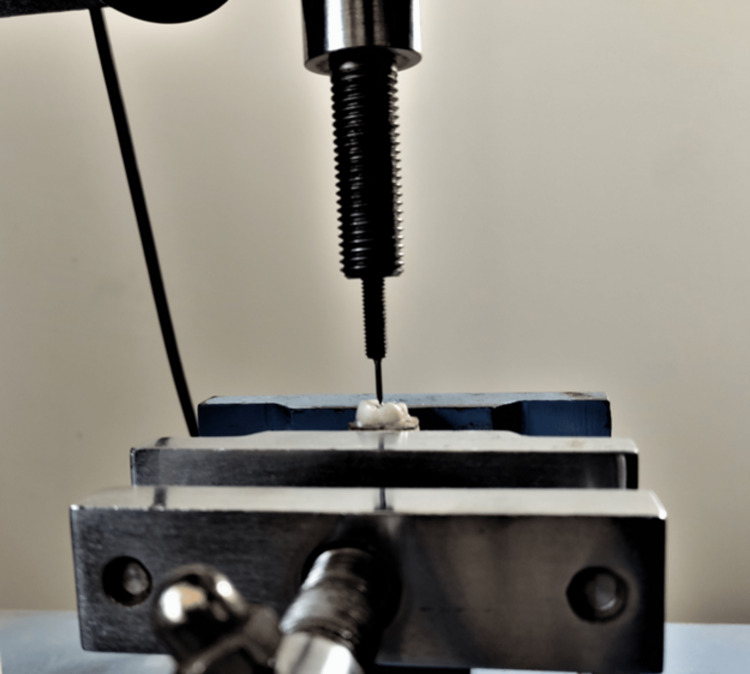
Fracture resistance test under a universal testing machine

Fracture resistance analysis (Figure 2) was carried out in the following groups: Group 1A: TAC+SFC; Group 1B: TAC+Type 9 GIC; Group 2A: CAC+SFC; Group 2B: CAC+Type 9 GIC; Group 3A: NAC+SFC; Group 3B: NAC+Type 9 GIC; Group 4A: TRAC+SFC; Group 4B: TRAC+Type 9 GIC. Teeth in SFC groups were restored with an incremental technique and light-curing equipment with Polywave technology and a power output of 600 mW/cm^2^ (Ivoclar Vivadent Bluephase N LED Dental Curing Light). After the cement was mixed according to manufacturer instructions, Type 9 GIC groups were filled using the plastic filling instrument. Samples of all the groups were embedded in self-cure acrylic resin up to 2 mm apical to the cementoenamel junction. These samples were then subjected to fracture load under a universal testing machine. The teeth were loaded at a 30° angle from the long axis of the tooth at the central fossa. An 8 mm diameter compressive head was used with a constant compressive force at a crosshead speed of 0.5 mm/min. The load at which the teeth fractured was determined by the testing machine's software and recorded in Newton.

Statistical analysis

The Shapiro-Wilk test for normal distribution was used to validate the data initially. Then, using the Tukey post hoc test and 2-way analysis of variance, the data was statistically analyzed. The significance level for this study was established at 5% (P < .05).

## Results

**Figure 3:**
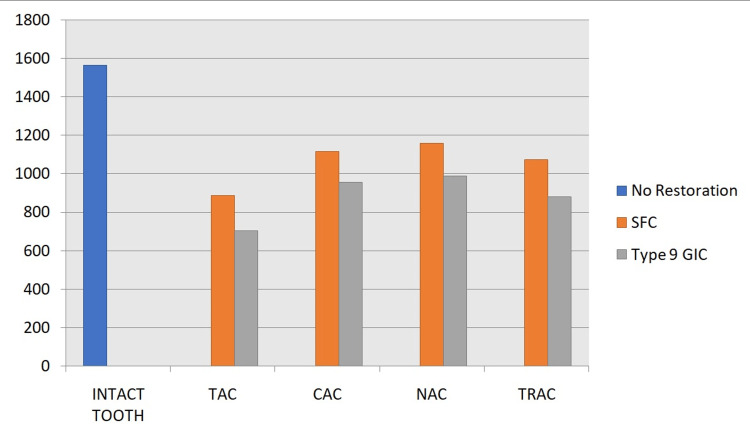
Graph Representing mean fracture resistance values of each group TAC: traditional access cavity; CAC: conservative access cavity; NAC: ninja access cavity; TRAC: truss access cavity; SFC: short fiber-reinforced composite, GIC: glass ionomer cement

The Fracture resistance values (N/mm^2^) obtained for each group presented as Mean Standard Deviation are shown in Table 1 and Figure 3. A two-way analysis of variance (P <.05) revealed that the control group samples (intact teeth) had significantly higher fracture resistance than the other test groups. There were significant differences in fracture resistance between teeth accessed using various access cavity designs (TAC, CAC, NAC, and TRAC) (P <.05). The TAC group has significantly lower fracture resistance values than the intact, CAC, NAC, and TRAC groups (P <.05), according to Tukey's post hoc analysis. Not much statistically significant difference was observed among the CAC, NAC, and TRAC groups (P >.05). Teeth restored with short fiber-reinforced composite had higher fracture resistance than teeth restored with Type 9 GIC (P < .01) independent of access cavity design.

**Table 1:** Fracture resistance values of each group presented as mean standard deviation TAC: traditional access cavity; CAC: conservative access cavity; NAC: ninja access cavity; TRAC: truss access cavity; SFC: short fiber-reinforced composite; GIC: glass ionomer cement

Groups (n=10)	Fracture Resistance Values (N/mm^2^) as Mean Standard Deviation
Intact tooth	1564±54.9
TAC+SFC	887±29
TAC+Type 9 GIC	703±96.2
CAC+SFC	1117±96
CAC+Type 9 GIC	956±153.1
NAC+SFC	1159±82
NAC+Type 9 GIC	989±103.2
TRAC+SFC	1075±96.02
TRAC+Type 9 GIC	879±154.8

## Discussion

During endodontic procedures, extensive preparation of endodontic access cavities diminishes tooth strength [1,2,12]. Furthermore, the elimination of hard tissue tends to increase cuspal flexure under occlusal load [13,14]. For that reason, a precise and condensed access design could optimize an endodontically treated tooth's prognosis [15]. Endodontic access cavities such as the Conservative access cavity (CAC), Ninja access cavity (NAC), and Truss access cavity (TRAC) have been recommended to minimize the fracture probability of endodontically treated teeth when compared to those accessed with a traditional access cavity (TAC).

The triumph of post-endodontic restorations is selecting an exemplary restorative modality to counterbalance the loss of coronal tooth structure [16]. There are various traditional methods for the reinforcement of endodontically treated teeth. Unfortunately, the majority of them compromise the minimal remaining tooth structure, resulting in root and/or crown fracture disposition [17]. 

Composite resins aren't widely used for large restorations or in greater stress-bearing areas despite advances in material sciences and with the idea of minimally invasive procedures due to polymerization shrinkage, high brittleness, poor fracture toughness, and the production of micro-cracks inside the tooth structure [18]. As a result, to increase mechanical properties, composite resins are reinforced with micro-glass fibers.

The effect of access cavity designs (TAC, CAC, NAC, and TRAC) in conjunction with post-endodontic restorative material (SFC, Type 9 GIC) on fracture resistance of endodontically treated teeth were investigated in this study. Mandibular molars were considered because vertical fractures are most prevalent in endodontically treated posterior teeth [19].

The root canals were endodontically prepared and filled before the fracture resistance test, and then post-endodontic restorations were done using everX posterior and high-strength posterior GIC, replicating the standard clinical methods. Nonetheless, the fracture methodology employed for in-vitro investigations does not accurately reflect intraoral settings in which failures occur mostly due to fatigue [20]. To circumvent variable outcomes due to varied operator competence, all specimen preparation procedures were performed by the same operator.

As per the current study's findings, there was a statistically significant difference in fracture strength between TAC, CAC, NAC, and TRAC (P < .05). Traditional access cavities (TAC) showed lower fracture resistance compared to modern access cavities. This could be due to the preservation of peri-cervical dentin in CAC, NAC, and TRAC. These findings corroborate past research that found no substantial variation in the fracture strength of these conservative access cavities [5,21].

Some investigations showed no significant difference in fracture strength between teeth treated with a conservative cavity or a traditional cavity [19,20]. A few studies have found that reducing endodontic cavity size using the conservative approach enhances the fracture resistance of teeth when compared to those treated by a traditional access cavity, enabling the preservation of residual dentin [5,22]. These contradictory findings might be attributed to differences in structured methodologies, such as the kind of tooth evaluated, the usage of restoration, the type of material employed for restorative treatments, and methodological difficulties associated with the fracture test design.

A contracted access cavity may have an impact on the efficacy of root canal treatment in addition to the association with fracture strength [20]. Primarily, it may affect the ability to locate root canals as well as the potential to thoroughly remove pulp tissue, debris, and necrotic material, which should be considered while designing the access cavity [5].

Groups that received short fiber-reinforced composite (GC everX Posterior) showed greater fracture resistance values when compared with the groups that received Type 9 posterior high strength posterior GIC (GC Gold Label IX). According to researchers, short fiber-reinforced composites can effectively withstand massive occlusal forces against fracture and strengthen the remaining tooth structure in endodontically treated teeth. This is because these composite resins were reinforced with short E-glass fiber fillers, which substantially improve the load-bearing capacity, flexural strength, and fracture toughness [23].

## Conclusions

Within the study's limitations, it can be inferred that preserving peri-cervical dentine has improved the fracture resistance in CAC, NAC, and TRAC. The fracture resistance of endodontically treated teeth has been greatly improved by using short fiber-reinforced composite (SFC). Hence, it can be a good choice for restoring teeth treated endodontically. Additional clinical research is needed to examine the effectiveness of instrumentation, challenges during endodontic treatments, and the long-term prognosis of endodontically treated teeth with CAC, NAC, and TRAC.
